# Galactofuranose antigens, a target for diagnosis of fungal infections in humans

**DOI:** 10.4155/fsoa-2017-0030

**Published:** 2017-06-01

**Authors:** Carla Marino, Adriana Rinflerch, Rosa M de Lederkremer

**Affiliations:** 1 Universidad de Buenos Aires, Consejo Nacional de Investigaciones Científicas y Técnicas, Centro de Investigaciones en Hidratos de Carbono (CIHIDECAR), Departamento de Química Orgánica, Facultad de Ciencias Exactas y Naturales, Pabellón II, Ciudad Universitaria, 1428 Ciudad Autónoma de Buenos Aires, Argentina; 2 Servicio de Dermatología, Dermatología Experimental, Hospital Italiano de Buenos Aires, C1199ACL, Ciudad Autónoma de Buenos Aires, Argentina

**Keywords:** biomarkers, diagnosis, fungal infections, galactofuranose, immune response, synthetic haptens

## Abstract

The use of biomarkers for the detection of fungal infections is of interest to complement histopathological and culture methods. Since the production of antibodies in immunocompromised patients is scarce, detection of a specific antigen could be effective for early diagnosis. D-Galactofuranose (Gal*f*) is the antigenic epitope in glycoconjugates of several pathogenic fungi. Since Gal*f* is not biosynthesized by mammals, it is an attractive candidate for diagnosis of infection. A monoclonal antibody that recognizes Gal*f* is commercialized for detection of aspergillosis. The linkage of Gal*f* in the natural glycans and the chemical structures of the synthesized Gal*f*-containing oligosaccharides are described in this paper. The oligosaccharides could be used for the synthesis of artificial carbohydrate-based antigens, not enough exploited for diagnosis.

## Lay abstract

D-Galactofuranose (Gal*f*) is the unit in polysaccharides and glycoconjugates of several pathogenic fungi that is recognized by the immune system. Since Gal*f* is not synthesized by mammals, it is an attractive candidate for diagnosis of infection. Since the production of antibodies in immunocompromised patients is scarce, detection of a specific antigen could be effective for early diagnosis. An antibody that recognizes Gal*f* is commercialized for the detection of aspergillosis. Chemically synthesized Gal*f*-containing oligosaccharides, reviewed in this paper, could therefore be used for the synthesis of artificial carbohydrate-based antigens and in diagnosis.

## Graphical abstract

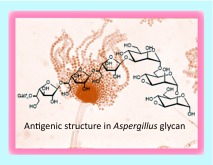

Effective serodiagnosis of systemic fungal infections is of increasing importance, particularly with regard to the identification of the infective organism. Early diagnosis, before infection is advanced, is still a challenge for efficient therapy. Clinical signs and culture detection methods for diagnosis of fungal infections are often slow, not specific and/or lack sensitivity [1,2]. Although they remain being the usual approaches for diagnosis of mycoses [3], lately, faster, nonculture-based methods have been developed, including immunoassays based on the presence of circulating galactofuranose (Gal*f*) antigens [4,5,6,7,8,9]. Since immunocompromised patients are not capable of producing enough antibodies for their detection in conventional serological tests, monoclonal antibodies have been obtained for the detection of specific antigens [5].

Aspergillosis is the most studied fungus-related disease and thus it will be preferentially referred. Invasive aspergillosis affects the lungs mainly in immunocompromised patients [10]. A review on molecular methods for diagnosis of invasive aspergillosis has been recently published [11].

The monosaccharide D-galactose (D-Gal) is very common in nature as a component of oligosaccharides and glycoconjugates [12]. It is interesting to remark that mammals are only able to synthesize the sugar in the pyranose configuration whereas some bacteria, fungi and protozoa have the unique enzyme UDP-Gal*p* mutase (UGM) that catalyzes the conversion of UDP-Gal*p* to UDP-Gal*f*, which is the donor of Gal*f* in the biosynthesis of Gal*f*-containing molecules [13,14]. The presence of Gal*f* was also reported in some nematodes [15,16]. In fungi, recombinant UGM from *Aspergillus fumigatus* was described [17]. Studies on the crystal structure and substrate-binding mechanism, revealed differences with the prokaryotic UGM [18]. The synthesis of the substrate UDP-Gal*p* is mediated by a UGE, which catalyzes the interconversion of UDP-Glc and UDP-Gal*p* [19]. In *A. fumigatus*, three genes encoding putative UGE have been reported [20], however, only one of them was required for the synthesis of Gal*f*-containing galactomannans and was essential for normal growth. Based on homology, a UGE was identified in *A. niger* [21]. A genetic and transcriptomic analysis of the cell wall of *A. niger* in response to the absence of Gal*f* was described [22].

Since UDP-Gal*f* is synthesized in the cytosol [23] and Gal*f* is incorporated in the Golgi, a UDP-Gal*f* transporter is required, and has been identified in *A. fumigatus* [24]. In this fungus, deletion of *UGM* caused attenuated virulence of the strain [18,25]. The Gal*f* transferases that incorporate Gal*f* into the glycans have been studied mainly in *Mycobacterium tuberculosis* [26,27]. In *Aspergillus* spp, a Gal*f* transferase involved in the biosynthesis of antigenic *O*-glycans was identified [28]. The glycans that contain Gal*f* are involved in immunological reactions [29,30,31], therefore they are envisioned as targets for diagnosis. This article aims to explore the perspective of methods based on synthetic sugar antigens for the production of monoclonal antibodies for serological detection of fungus-specific antigens.

## β-galactofuranosyl structures in human infective fungi

Gal*f* is mainly present in the β-configuration in fungi, many of them involved in mammal infections. It is also present in plant pathogenic fungi, but these will not be discussed in the present review.

Fungi that infect mammals and contain glycans with Gal*f* are listed in Table 1. Gal*f* is usually present as terminal sugar, linked to each other by β(1→6) or β(1→5) linkages or branching a mannan core at the *O*-2 or *O*-3 position of mannose.

**Table 1.** **Structural units containing β-galactofuranose in mammal-pathogenic fungi.**

**Organism**	**Structure^†^**	**Study**	**Ref.**
*Aspergillus*	Gal*f*(β1→5)Gal*f* Gal*f*(β1→3)Man Gal*f*(β1→6)Man Gal*f*(β1→2)Man Gal*f*(β1→5)Gal*f*(β1→6)Man(α1→6)Man	Latgé Tefsen *et al*. Fontaine *et al*. Jin Leitã *et al*.	[29,32,33,34,35]
*Neosartorya*	[→6)Gal*f*(β1→5)Gal*f*(β1→5)Gal*f*(β1	Leal *et al*.	[36]
*Neotestudina rosatii*	Glc(α1→2)Gal*f*(β1→6)Gal*f*β Glc(α1→2)Gal*f*(β1→2)Man	Leal *et al*.	[37]
*Fusarium*	Gal*f*(β1→6)Gal*f*	Ahrazem *et al*. Wiedemann *et al*.	[31,38]
*Trichophyton*	Gal*f*(β1→3)Man	Ikuta *et al*.	[39]
*Malassezia*	Gal*f*(β1→6)Gal*f*	Shibata *et al*.	[40]
*Sporothrix schenckii*	Gal*f*(β1→6)Gal*f* Gal*f*(β1→2)Man	Mendonça-Previato *et al*.	[41]
*Cladosporium herbarum*	Gal*f*(β1→6)Gal*f*(β1→6)Gal*f*(β1→2)Man(α1→6)Man	Swärd-Nordmo *et al*.	[42]
*Paracoccidioides*	Gal*f*(β1→6)Man(α1→2) Man	Almeida *et al*. Levery *et al*. Ahrazem *et al*.	[30,38,118]
*Neotestudina rosatii*	Glc*p*(α1→2)Gal*f*(β1→6)Gal*f*β	Leal *et al*.	[37]
*Fonsecaea pedrosoi*	Glc(α1→2)Gal*f*(β1→6)Man(α1→2) Man	Shibata *et al*.	[44]
			
			

^†^When configuration is not indicated, it corresponds to the pyranosyl configuration. All the sugars belong to the D-series.

In 1985, Bennet *et al*. showed that galactofuranosyl groups are immunodominant in *A. fumigatus* galactomannan (GM) [45], and accordingly, Gal*f*-deficient mutants of *A. fumigatus* display an attenuated virulence [25].

The cell walls of *A. fumigatus* have at least four different types of molecules containing Gal*f*, which are important for cell wall integrity (Table 1) [29,32,33,34,35,46,47]. Gal*f* units were identified in *O*- and *N*-linked chains of glycoproteins and also in glycoinositolphosphorylceramides [48]. The glycoinositolphosphorylceramides with Gal*f*(β1→6) linked to a mannose core were highly immunogenic.

*Neosartorya* spp., teleomorph of *A. fumigatus*, are a cause of invasive disease in immunocompromised patients [49]. Acute respiratory distress syndrome has been attributed to *N. udagawae* [50]. The structure [→6)-Gal*f*(β1→5)-Gal*f*(β1→5)-D-Gal*f*(β1→] has been determined in polysaccharides of some species of *Neosartorya* [36].

*Fusarium* is a pathogen of plants, but some species are pathogenic for humans, particularly *Fusarium verticillioides* and *F. proliferatum* [51]. *Fusarium* species cause a broad range of opportunistic infections in humans. In healthy individuals, the most common clinical manifestations are onychomycosis, skin infections and keratitis, whereas in immunocompromised patients disseminated infections with multiple necrotic lesions may occur [52].

It is important to discriminate *Aspergillus* from *Fusarium* infections since both differ in their response to common antifungals. Using a combination of two antibodies, both species could be differentiated by immunohistology (see below) [31].

*Trichophyton* spp., in particular *T. mentagrophytes* and *T. rubrum* cause chronic dermatophyte infections, often associated with infection of the nails (onychomycosis) [53]. Although they first infected animals, they adapted to infect humans and are now considered a major health problem [54]. Cell wall antigens secreted by the fungus may diffuse into the dermis and establish the infection, due to immunosuppressive effects. Impairment of lymphocyte proliferation was shown [55]. One of the major cell wall components secreted to the medium is a GM. Structure determinations showed that Gal*f* terminal units are β(1→3) linked to a mannan core. Polygalactofuranosyl chains, similar to those produced by *A. fumigatus*, have not been found in *Trichophyton* [39]. Accordingly, a monoclonal antibody against the GM of *A. fumigatus* showed very low cross-reactivity with exoantigens from cultures obtained from clinical specimens [56]. Glycosylphosphatidylinositols labeled with [^3^H]-galactose and [^3^H]-mannose were biosynthesized by membrane preparations of *T. rubrum*. The lability to acid of the galactose suggested its furanosic configuration [57].

A linear chain of β1→6 linked Gal*f* attached to a small mannan was also found in polysaccharides of *Malassezia* spp [40]. *Malassezia* spp. are human pathogens responsible for skin diseases and they are also associated with catheter-related fungaemia [58].

*Sporothrix schenckii* is the agent of sporotrichosis in humans and animals, producing skin and subcutaneous lesions. It is present in all continents, especially in tropical and subtropical areas [59,60]. In an early work, Gal*f*-containing polysaccharides were isolated from the supernatants of *S. schenckii* cultures [41]. It was found that a mannan core is substituted by β-Gal*f* chains which are responsible for cross-reactions with other fungal antigens. Apparently, no further studies on this GM were described. In turn, peptidorhamnomannans with both *N*- and *O*-linked carbohydrate chains as the immunodominant structures were obtained from extracts of the cell walls [61]. Later studies characterized a peptidorhamnomannan (Gp70) isolated from the yeast phase of *S. schenckii* as an adhesin involved in the host–pathogen interaction, but Gal*f* was not reported as a constituent of this glycoprotein [62].

*Cladosporium* spp. are mainly plant pathogens but some may trigger allergic reactions in sensitive individuals. Prolonged exposure to a high concentration of spores may produce chronic asthma. A glycoprotein named Ag-54, which contains 80% carbohydrate, was an allergen purified from *C. herbarum*. Structural studies showed chains of β1→6 linked Gal*f* bound to *O*-2 of 1→6 linked mannoses (Table 1) [42].

*Paracoccidioides brasiliensis* is endemic to regions of Latin America, causing a mycosis which spreads from the lungs to many organs and if not treated could be fatal [63]. Paracoccidioidomycosis (PCM) is commonly diagnosed by identifying budding yeast cells in biological fluids or histologically [64]. Glycolipids extracted from yeast and mycelium forms of *P. brasiliensis* reacted with sera from patients. The antibodies were directed mainly to the galactofuranosyl units in the glycolipids [65]. A review on glycolipids in fungi and trypanosomatids discusses the role of Gal*f* in the infectivity [66]. However, the main diagnostic antigen of *P. brasiliensis* is the exocellularly secreted glycoprotein gp43, which contains a terminal Gal*f* unit attached (β1→6) to a mannose in an *N*-linked oligosaccharide chain [30].

Antigenic Gal*f* is usually present as an exposed terminal sugar in the glycan (Table 1). However, in some fungi an internal Gal*f* is present. This is the case of *Neotestudina rosatii*, from which three polysaccharides containing the units Glc(α1→2)Gal*f* (β1→6)Gal*f*- and Glc(α1→2)Gal*f* (β1→2)Man linked to a mannan were extracted [37].

*Neotestudina rosatii* is one of the etiologic agents of subcutaneous infections in humans. This species occurs with other fungi causing eumycetoma, and its taxonomic classification is uncertain [67].

A glycoprotein with *N*- and *O*-glycans was extracted from *Fonsecaea pedrosoi*, the etiologic agent of chromoblastomycosis. The hexasaccharide containing internal Gal*f* (Table 1) was the main *O*-linked chain [44,68]. Specific antibodies against *F. pedrosoi* were strongly inhibited by the hexose, but the contribution of the internal Gal*f* to the antigenicity was not evaluated. Probably, the main epitope in *F. pedrosoi* is the external α-Gal*p*, a recognized antibody in humans [69].

In the previously mentioned examples, Gal*f* is present in the β-anomeric configuration. However, the less common α-Gal*f* has also been found in some organisms, such as, *P. brasiliensis*. Interestingly, the configuration of the sugar depends on the phase, whereas β-Gal*f* is present in the yeast form infecting the mammal, α-Gal*f* is a constituent of the mycelial GM (Table 2) [38,70].

**Table 2.** **Structural units containing α-galactofuranose in mammal-pathogenic fungi.**

**Organism**	**Structure^†^**	**Study**	**Ref.**
*Paracoccidioides brasiliensis*	Gal*f* (α1→6) Man(α1→2) Man	Ahrazem *et al*. San-Blas *et al*.	[38,70]
*Histoplasma capsulatum*	Gal*f* (α1→6) Man (α1→2 or 6)	Barr *et al*.	[71]
*Aspergillus niger*	Galf (α1→2) Man	Takayanagi *et al*.	[72]

^†^All the sugars belong to the D-series.

Gal*f* was also found in glycoinositolphospholipids obtained from the yeast phase of *Histoplasma capsulatum*, the causative agent of histoplasmosis, an endemic mycosis [71]. *Histoplasma capsulatum* is a dimorphic fungus which grows in the soil as a filamentous mycelium, but it converts to a yeast-like form in the tissues of infected animals. The purified glycoinositolphospholipids were shown to react with sera from histoplasmosis patients [71]. The authors tentatively assigned the α-configuration for the Gal*f* from the yeast form, in contrast with the configuration found for the Gal*f* in the same phase in *P. brasiliensis*. The configuration of galactofuranosides is unequivocally assigned by NMR spectroscopy [12], which was not used by Barr *et al*. in 1984 [71]. The glycosphingolipids from the mycelial phase were not described.

In *A. niger*, α-Gal*f*(1→2) linked to mannose was characterized [72]. Although this species is less pathogenic for humans than *A. fumigatus*, it could produce allergic reactions in high concentrations.

The antigenic properties of β-galactofuranosides are well known [29,30,31] but the immunological role of α-Gal*f* was apparently not described. The differences in the Gal*f* configuration in the galactomannans of the infective *P. brasiliensis* and *A. fumigatus* could explain the low cross-reactivity in serological tests using a specific antibody [73].

## Structural analysis of Gal*f*-containing polysaccharides or glycoconjugates

In earlier works, the identification of Gal*f* units in glycoconjugates was performed using chemical methods. Taking advantage of the greater lability of furanosic linkages with respect to the pyranosic bonds, terminal Gal*f* units may be selectively released from the molecule by mild acid hydrolysis, separated by chromatographic techniques or by dialysis, depending on the MW of the remaining degraded molecule, and then analyzed by chromatographic methods or even GC–MS. Also, the exocyclic chain of terminal Gal*f* could be selectively oxidized by periodate under mild conditions, and upon reduction with NaBH_4_ converted to arabinofuranose, which may be identified after acid hydrolysis by GC–MS. Application of these methods is exemplified in the report on the determination of the structure of glycolipids of *P. brasiliensis* [65]. Information on the substitution position of the galactofuranosyl linkages is provided by methylation analysis [74].

NMR has also been useful for the identification of Gal*f* units in oligosaccharides. ^1^H NMR spectra usually show deshielded anomeric signals for Gal*f* units, compared with those of pyranosic units. Particularly useful is the ^13^C NMR spectroscopy because the furanosyl ring has a specific signal pattern, easily distinguishable from the pyranosyl analog. For β-Gal*f* units, signals corresponding to the anomeric carbon, and C-2 and C-4 resonate significantly deshielded in comparison with pyranosic signals. In addition, α-Gal*f* has also a particular pattern. Advances in NMR spectroscopy have facilitated the characterization of oligosaccharides. Thus, bidimensional techniques allow not only the identification of the monosaccharides and their anomeric configuration but also to stablish the position of substitution and sequences [35,39,75].

## Chemically synthesized oligosaccharides of antigenic glycans

The use of chemically synthesized oligosaccharides of defined structure avoids the disadvantages of glycans obtained from natural sources, such as, the difficulty of isolating and purificating sufficient quantities, and problems associated with their heterogeneity. Also, more specificity may be achieved by using a neoantigen. Following this line, a biotinylated tetrasaccharide with Gal*f*(β1→5)Gal*f* linkages was synthesized (Table 3A; n = 2). This synthetic antigen was recognized by the monoclonal antibody EB-A2 against *Aspergillus* [76].

**Table 3.** **Structures with galactofuranose units found in fungal glycans which have been chemically synthesized.**

**Structure**			**Study**	**Ref.**
**A**	n = 0 n = 0–5 n = 2	R = H, Bn R = CH_3_ R = C_8_H_17_ R = C_2_H_5_ R = H, Pr R = H, allyl R = (CH_2_)_2_CHNH_2_COOH R = arm–biotin	van Heeswijk *et al*. Lederkremer *et al*. Completo and Lowary Pathak *et al*. Gurjar *et al*. Sugawara *et al*. Veeneman *et al*. Cattiaux *et al*.	[26,76,77,78,79,80,81,82]
**B**	n = 0 n = 4	R = CH_3_ R = C_8_H_17_ R = allyl R = allyl	Marino *et al*. Completo and Lowary Euzen *et al*. Pathak *et al*. Splain and Kiessling Zhang *et al*.	[26,79,83,84,85,86]
**C**	R = decenyl R = octyl		Gandolfi-Donadio *et al*. Completo and Lowary	[26,87]
**D**	R = decenyl R = octyl R = CH_3_		Gandolfi-Donadio *et al*. Completo and Lowary Deng *et al*.	[26,87,88]
**E**	R = H R = decenyl R = (CH_2_)_8_CO_2_CH_3_		Marino *et al*. Baldoni and Marino Tsui *et al*.	[89,90,91]
**F**	R = CH_3_		Fu *et al*.	[92]

In Table 3, the structures of Gal*f*-containing oligosaccharides, which have been chemically synthesized and are part of fungal glycans, are shown. Most efforts have been directed to the synthesis of oligosaccharides containing Gal*f*(β1→5)Gal*f*, the antigenic unit in *Aspergillus* [26,77,78,79,80,81,82].

**Figure 1.**
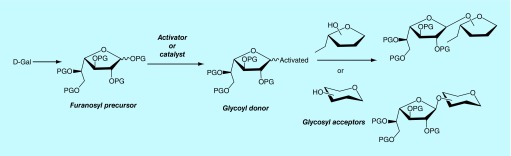
**General strategy for the synthesis of Gal*f*-containing molecules.**

Several chemical syntheses for the Gal*f*(β1→6)Gal*f* disaccharide have been reported (Table 3B) [26,83,84,85]. This is the minimal epitope in *Fusarium* glycans [31]. A hexasaccharide with the same linkages was synthesized [86]. The two trisaccharides containing both types of linkages (Table 3C & D) were prepared [26,87,88] because of their importance as repeating units in the arabinogalactan of *M. tuberculosis* [93], but is also present in a polysaccharide extracted from *Neosartorya* [36]. Gal*f* is frequently linked to mannose forming galactomannans. The synthesis of the disaccharide Gal*f*(β1→3)Man (Table 3E) [89,90,91] and the more complex heterotetrasaccharide (Figure 1F) [92], which was indicated as the minimal epitope in the mycelial cell wall of *A. fumigatus* [35], were described.

The synthesis of Gal*f*-containing molecules requires the efficient preparation of derivatives of D-Gal in the furanosic configuration, free from the pyranosic forms, as precursors of Gal*f* units in the target molecules. Furthermore, efficient glycosylation methodologies and the consequent availability of galactofuranosyl donors as well as conveniently substituted derivatives as glycosyl acceptors are required (Figure 1) [27,94,95,96].

Most sugars lead by conventional methods to pyranosic derivatives, which are thermodynamically more stable. However, in conditions under which other monosaccharides lead mainly to pyranosic products, large proportions of galactofuranosic derivatives can be obtained from galactose. This is the case of the traditional Fisher glycosylation, which yields a mixture of furanosic and pyranosic glycosides that must be separated by chromatography [27]. An example may be found in the preparation of the pentenyl galactofuranosides which were used as precursors in glycosylation reactions [97].

**Figure 2.**
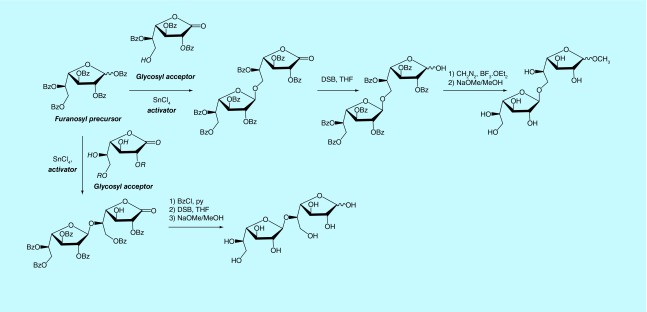
**Synthesis of disaccharides present in fungal galactomannans.** DSB: Disiamylborane; THF: Tetrahydrofuran.

The peracylated derivatives are commonly used as precursors, particularly penta-*O*-benzoyl-Gal*f* (BzGal*f*) that may be obtained as crystalline products, in one step, from galactose [98]. Glycosylation of BzGal*f* promoted by a Lewis acid affords the β-galactofuranosides, as a result of anchimeric assistance. In Figure 2, an example for the synthesis of the natural disaccharides Gal*f*(β1→5)Gal*f* [83] and Gal*f*(β1→6)Gal*f* [78] is shown.

One of the most popular glycosylation procedures is the trichloroacetimidate method [99], which is compatible with acid-sensitive acceptors and was used first for the synthesis of Gal*f*(β1→3)GlcNAc [100]. As this is an area of active research, several more methods have been designed and extensively reviewed [26,95,96]. The synthetic oligosaccharides could be precursors for artificial antigens to be used for diagnosis [101] or for the construction of synthetic vaccines [102,103].

## Immunologic detection of Gal*f*-containing molecules

A monoclonal antibody, which recognized Gal*f*(β1→5)Gal*f* epitopes was produced by immunizing rats with an extract of *A. fumigatus* mycelia. The antibody called EB-A2 also detected the GM of other *Aspergillus* species, using an ELISA assay [104,105]. It was commercialized as Platelia™ *Aspergillus* ELISA (Bio-Rad Laboratories and Sanofi Diagnostics, CA, USA). The specificity and sensitivity of the assay were reviewed [106]. Multivalent gold nanoparticles carrying Gal*f* were recognized by this antibody [107]. The presence of GM in circulation depends on the manifestation of the disease. For instance, negative results are commonly obtained for the detection of GM in sera of allergic bronchopulmonary aspergillosis. Reviews on diagnosis of aspergillosis have been published [108,109,110]. Apparently, this is the only commercialized test based on detection of Gal*f*. It was shown that EB-A2 detects GM in supernatants of several *Fusarium* species [111,112]. However, Wiedemann *et al*. described a novel monoclonal antibody, AB 135–8, which recognizes Gal*f* in *Fusarium* but as part of a different antigen [31]. In fact, the structural unit Gal*f*β(1→6)Gal*f*, was characterized in a polysaccharide extracted from *Fusarium* [75] but it was not reported in *Aspergillus* (Table 1). The same antigenic disaccharide was found in *Malassezia* galactomannans, which did not react with the EB-A2 antibody against *A. fumigatus*, but apparently was not tested with AB 135–8 against *Fusarium.* Antibodies against *M. furfur* did not react with galactomannans of *A. fumigatus*, *T. rubrum* or *F. pedrosoi*, confirming the presence of a different linkage for the Gal*f* units, but apparently these were not tested with AB 135–8 against *Fusarium* [40].

The Platelia test was negative for yeast and mold forms of *S. schenckii* [113]. Controversial results were reported for *Cryptococcus neoformans*. While a positive test was reported for a patient with *C. meningitis* [114], a later report revealed no cross-reaction with culture extracts, purified polysaccharides, clinical specimens and specimens from animals following experimental infection [115]. These results are explained by the composition of the cell wall of *C. neoformans*. Although galactoxylomannan is a constituent of the cell wall, and D-Gal is its major component, this sugar is mainly in the pyranosic configuration, with only 2% tentatively assigned to Gal*f*, considering a small peak in the ^13^C NMR spectrum [116], which is as explained above, is not enough to guarantee the configuration. More recently, it was demonstrated that Gal*f* is not required for growth or virulence of *C. neoformans* [117].

In *P. brasiliensis*, Gal*f* is the immunodominant unit in a glycosylinositolphosphoryl ceramide [43], however, the main diagnostic antigen is the glycoprotein gp43. Studies of *N*-deglycosylation gave rise to a protein of 38 kDa, which strongly reacted with sera from patients with PCM [118]. Also, recombinant gp43 isoforms as *N*-mannosylated proteins were expressed in the yeast *Pichia pastoris* and showed good specificity for detection of PCM in sera [119]. These results indicate the presence of other epitopes in gp43, although the cause of cross-reactivity with sera from patients with other mycoses would be the terminal Gal*f* [118 120]. Immunodiagnosis of gp43 in PCM using a latex test was recently described [73].

In the previous examples, Gal*f* is present in the galactomannans in the β-anomeric configuration. A few examples of α-Gal*f* may be seen in Table 2. The antigenic properties of α-Gal*f* were apparently not described. As expected, isolates from *H. capsulatum* in yeast and mold phases were not recognized by the monoclonal antibody against the *Aspergillus* GM [113]. However, it was reported that some patients with disseminated histoplasmosis gave positive results with this antibody [121].

## Conclusion & future perspective

Gal*f* is an antigenic structural unit in many infectious fungi, which is not biosynthesized by humans. The preparation of synthetic neoglycoconjugates containing β-Gal*f* for the diagnosis of some extended mycoses as aspergillosis is a field worth to explore. The synthetic antigens would help define the structure of the corresponding epitopes and in a more ambitious project they could be the starting line for the synthesis of carbohydrate-based vaccines [102]. In reviewing the natural structures, it is challenging to understand why in some dimorphic fungi the commonly found β-Gal*f* changes the anomeric configuration to α-Gal*f*, in the transition between phases. The α-Gal*f* transferases necessary for the construction of the linkages in some galactomannans have not yet been described.

**Executive summary**

**Biomarkers**

- Diagnosis of fungal infections and identification of the causative agent is important for the selection of an accurate therapy. The use of biomarkers may provide faster results and complement culture and histological methods.

**Carbohydrate-based markers**

- Galactofuranose (Gal*f*) is an attractive candidate as a biomarker for diagnosis of infections, since it is the antigenic epitope in glycans of several pathogenic fungi and is not biosynthesized by mammals. An ELISA assay based on the EB-A2 antibody that detects the Gal*f* in the GM of *Aspergillus* is commercialized. The different linkages of the Gal*f* to other sugars in the fungal carbohydrates may provide specificity, a line of research that needs further studies.

**Chemically synthesized oligosaccharides of antigenic glycans**

- The impressive advance achieved in the chemical synthesis of the natural oligosaccharides encourages their use as specific antigens.
